# From Analysis to Assessment: Machine Learning for Non-Target Screening of Pollutants Using Chromatography Coupled with (Ion Mobility) Mass Spectrometry

**DOI:** 10.3390/toxics14040322

**Published:** 2026-04-13

**Authors:** Dongshan Lin, Zhenyue Wang, Jiaqi Liao, Nan Li, Xiaolei Li

**Affiliations:** College of Ocean Science and Meteorology, Guangdong Ocean University, Zhanjiang 524088, China; ldsh313@stu.gdou.edu.cn (D.L.); wangzhenyue@stu.gdou.edu.cn (Z.W.); liaojiaqi@stu.gdou.edu.cn (J.L.); nli0417@163.com (N.L.)

**Keywords:** machine learning, non-target screening, ion mobility, mass spectrometry

## Abstract

The growing diversity of anthropogenic chemicals in the environment far exceeds the scope of routine analytical monitoring. Non-target screening (NTS) using high-resolution mass spectrometry (HRMS) has thus emerged to discover unknown organic contaminants. Liquid or gas chromatography (LC/GC) coupled with ion mobility–mass spectrometry (IM-MS) further enhances NTS by providing multidimensional, structurally informative data. Machine learning (ML) offers a powerful solution by efficiently processing high-dimensional data and uncovering patterns. Both supervised and unsupervised learning approaches show strong potential to streamline labor-intensive processes. This review provides an overview of key ML algorithms and representative workflows in LC/GC-(IM-) MS-related NTS, followed by a critical synthesis of recent advances in ML-enabled applications across the entire NTS procedure, from sample analysis to data acquisition, and ultimately risk assessment. Continued advances in ML are expected to transform NTS into a more efficient and robust tool for risk assessment.

## 1. Introduction

A vast number of chemicals are manufactured, used, and ultimately released into the environment each year. For instance, the U.S. Toxic Substances Control Act (TSCA) Inventory lists over 86,000 chemicals [1]. Many of these anthropogenic substances undergo environmental degradation or human activities, resulting in the production of massive known and unknown metabolites. However, only 31 chemicals or chemical groups are currently regulated as persistent organic pollutants (POPs) under the Stockholm Convention [2]. Thousands of additional compounds have been reported to pose potential environmental and human health risks [3,4].

To address the need for broad chemical monitoring, non-target screening (NTS) approaches have been developed to detect and characterize unknown organic pollutants [5]. NTS is an integrated procedure that starts with sampling, and its ultimate goal is to provide comprehensive data for risk assessment. Despite significant progress, NTS faces major challenges. First, the chemical space of both known and unknown compounds is huge. Second, many of these analytes occur at very low concentrations (often <ng/mL or <ng/g), complicating detection and identification. Moreover, predicting transformation products remains a challenge due to complex and dynamic environmental conditions, further hindering confident identification in NTS workflows [6].

High-resolution mass spectrometry (HRMS) coupled with liquid chromatography (LC) or gas chromatography (GC) has become the primary analytical approach for NTS, owing to its exceptional mass accuracy and resolving power for distinguishing closely related ions. In recent years, ion mobility spectrometry (IMS) has gained renewed attention due to its ability to separate isomers and isobars, and thereby substantially enhancing the overall resolving power when integrated with HRMS. In IMS, ions migrate through a neutral buffer gas (typically nitrogen or helium) under the influence of an electric field. Their drift behavior, termed ion mobility, is determined by ion size, shape, and charge state, and is commonly expressed as the collision cross section (CCS, Å^2^). This structure-dependent physicochemical descriptor provides an additional dimension of separation for HRMS analysis. Notably, particular isomeric species that share identical masses but differ in ion mobility can exhibit distinct toxicities [7]. The special relationship between CCS and mass across different chemical groups has facilitated chemical identification [8]. Information from chromatography, mass spectrometry, and ion mobility can complement each other in NTS, where a single analytical dimension is usually insufficient for confident characterization of unknowns.

When broad-spectrum extraction procedures and soft ionization sources are employed, thousands of non-target features may be detected within a single sample [9]. A significant bottleneck arises from the data interpretation process, which continues to rely heavily on manual or semi-manual peak picking. This approach is labor-intensive, time-consuming, and requires extensive operator expertise, limiting throughput and reproducibility. In current practice, widely used public software tools such as ProteoWizard [10], XCMS [11], MS-DIAL [12], and MZmine [13,14] are commonly used data handling approaches, which include functions such as raw data conversion, peak detection, deconvolution, feature alignment, and preliminary annotation. In addition, classical chemometric approaches or algorithms, including PARAFAC [15,16] (the algorithm PARADISE [17] used), and MCR [18] (the algorithm MCR-ALS [19] and ROIMCR [20] used) remain important for analyzing structured LC/GC-MS data, particularly for resolving overlapping signals and extracting chemically interpretable information from raw analytical data.

To better leverage these rich datasets and accelerate data interpretation, machine learning (ML), a branch of artificial intelligence, has gained substantial momentum, supported by the increasing accessibility of open-source tools in Python and R. ML enables automated data interpretation through statistical modeling and pattern recognition. In supervised learning, the most widely used ML paradigm, algorithms are trained on input–output pairs to learn complex relationships and generate accurate predictions for previously unseen inputs [21]. Supervised ML has already been applied to a broad range of data-intensive challenges and is beginning to provide new insights into NTS and IMS-enhanced HRMS analysis. In contrast, unsupervised learning operates on unlabeled datasets, allowing algorithms to identify inherent structures or generate representations without predefined categories. Although highly relevant for high-dimensional chemical datasets, unsupervised ML remains underexplored in the context of NTS. Semi-supervised learning combines a small amount of labeled data with a much larger pool of unlabeled data, making it particularly valuable when obtaining high-quality labels is costly, time-consuming, or analytically challenging. This approach leverages the strengths of both supervised and unsupervised learning while reducing the dependency on labor-intensive annotation.

In this review, we first introduce commonly used ML algorithms and their workflow considerations. We then summarize recent applications of ML in ion mobility and mass spectral prediction, automated peak annotation, and the discovery of unknown environmental pollutants. Additional NTS-relevant applications, including pollutant source tracking and metabolite prediction using MS data, are also discussed. Finally, we highlight current limitations, future opportunities, and the broader implications of integrating ML into non-target screening. Our goal is to provide a clear, accessible overview of ML principles and recent advances in (ion mobility) mass spectrometry for practitioners in the NTS community, while encouraging collaboration between environmental scientists and computer scientists to develop more powerful solutions for the interpretation of complex HRMS datasets.

## 2. Machine Learning Workflow Currently Used in NTS

### 2.1. Common Learning Algorithms Used in NTS

Modern computer science has seen rapid advances in ML, leading to many algorithms applicable to chemical data analysis. Although the mathematical underpinnings of these algorithms are often complex, a detailed theoretical treatment is not required for their effective use in NTS. Therefore, this section provides a concise overview of the ML approaches most relevant to NTS and IMS-MS workflows.

#### 2.1.1. Supervised Learning Algorithms

Supervised ML encompasses methods that learn predictive relationships between input features and known output labels [22]. Among supervised ML methods, the two most common applications are classification, which assigns samples to discrete categories, and regression, which predicts a continuous response variable. Many supervised algorithms can be applied to both tasks. Decision trees, random forests, gradient boosting machines, support vector machines, and artificial neural networks are widely used across classification and regression problems in HRMS-based environmental analysis. Their flexibility, nonlinear modeling capabilities, and ability to incorporate high-dimensional features make them well-suited to the large, complex datasets generated in NTS studies.

A decision tree (DT) predicts an outcome by passing input features through a sequence of decision nodes. Each node applies a threshold to split the data, forming a hierarchical “tree” that terminates in a final class assignment or numerical prediction. Random forests (RFs) extend DTs by building an ensemble of many trees, each trained on slightly different subsets of the data. The final prediction is obtained by aggregating the outputs of these trees, thereby significantly improving performance and reducing information loss relative to a single DT. Because these “tree-based” methods learn directly from examples of known samples, they can be used to classify new samples whose feature values are provided. However, their predictions are limited to the categories included in the training data; they cannot reliably classify samples from entirely novel sources. A notable advantage is that DT and RF models can be trained using standard desktop computers, making them accessible for routine use [23]. Support vector machines (SVMs) provide another supervised approach by constructing a boundary (hyperplane) that best separates two classes in feature space. Although highly effective for nonlinear data, traditional SVMs are primarily suited for binary classification problems [24].

Several statistical learning methods are also frequently used. Partial least-squares (PLS) regression reduces high-dimensional input data to a smaller set of latent variables while maximizing their ability to predict the response. This makes PLS particularly useful when the number of predictors is large relative to the number of samples. Multivariate logistic regression (MLR) models the probability that a sample belongs to a particular class, using maximum likelihood estimation to determine the optimal model parameters. Because logistic regression is susceptible to overfitting, especially with a large number of predictors, a regularization step (RMLR) is commonly applied. MLR performs well in both binary and multi-class classifications. Simpler distance-based methods, such as k-nearest neighbors (KNN), classify or cluster samples by comparing their distance to known examples. While they are generally less potent than RFs for complex datasets, they are less prone to overfitting and remain useful for exploratory analysis.

Artificial neural networks (ANNs) are computing models inspired by the structure of biological neural systems. They consist of an input layer, one or more hidden layers, and an output layer. Information is processed through interconnected nodes (neurons), each with associated weights and biases learned during training. Unlike tree-based or regression algorithms, ANNs do not rely on explicitly defined decision rules; instead, they understand complex relationships directly from the data. Graph Neural Networks (GNNs) are a specialized extension of ANNs designed to operate on graph-structured data. GNNs have demonstrated significant potential in handling graph-structured data, opening innovative possibilities for leveraging fragmentation trees in model development. By utilizing hierarchical information propagation, GNNs can efficiently capture and integrate the intricate details contained within fragmentation trees. Consequently, designing GNN-based models that incorporate fragmentation trees could facilitate end-to-end predictions of compound bioactivity directly from HRMS data, marking a transformative shift in traditional approaches to model construction.

ANNs form the foundation of deep learning (DL) methods, of which convolutional neural networks (CNNs) are a prominent example. CNNs apply mathematical convolution operations to extract patterns from grid-structured data and have recently gained attention in NTS research. Despite their flexibility and strong predictive capability, ANN-based models often require substantial computational resources, and can be performed in supervised (most common), semi-supervised, and unsupervised ways.

In regression tasks, the goal is to predict a continuous output variable by modeling the relationship between features and outcomes. Gradient boosting (GB) algorithms build models sequentially, with each new decision tree attempting to correct the errors made by previous ones. By iteratively minimizing a loss function, GB methods significantly enhance the predictive performance of single decision trees and are widely regarded as robust approaches for both regression and classification.

#### 2.1.2. Unsupervised Learning Algorithms

In addition to supervised methods, several unsupervised learning techniques play essential roles in non-target screening, where the aim is to reveal structure in the data without predefined labels.

Principal component analysis (PCA) is one of the most commonly used unsupervised methods for dimensionality reduction and exploratory data analysis. PCA projects the original data onto new coordinate axes that capture the maximum variance in the dataset. In NTS, PCA is used to describe major variance patterns across features in a training set and to identify highly variable features associated with anomalous or outlier samples. Because PCA primarily highlights dominant trends, subtle variations in the data may be overlooked.

Non-negative matrix factorization (NMF) is another unsupervised technique reported to be applied to spectral or compositional datasets. By decomposing a data matrix into two non-negative component matrices, NMF identifies underlying patterns or source contributions in a way that is often more interpretable than PCA, particularly when variables represent intensities or abundances that cannot be negative. NMF is therefore valuable for uncovering latent structures in complex HRMS datasets.

k-Means Clustering is a widely used unsupervised clustering algorithm that partitions data into k clusters by minimizing the within-cluster variance. It iteratively assigns samples to the nearest cluster centroid and updates the centroid positions until convergence. Although simple and computationally efficient, k-means assumes spherical clusters and may be sensitive to the choice of k and initial centroids. In NTS, it is often applied to group features with similar mass spectral or chromatographic characteristics.

Hierarchical clustering groups samples based on their similarity in a stepwise manner, either by progressively merging smaller clusters or splitting larger clusters. The results are typically visualized as a dendrogram, which reveals the nested structure of relationships among samples. It is widely used in NTS to explore chemical similarity patterns and identify clusters of related features.

Density-Based Spatial Clustering of Applications with Noise (DBSCAN) is a density-based clustering algorithm that identifies clusters as regions of high point density separated by areas of low density. It can detect clusters of arbitrary shape and is particularly effective at identifying outliers or noise points. In NTS data analysis, DBSCAN helps find groups of chemically related features and detect anomalous signals.

#### 2.1.3. Semi-Supervised Learning Algorithms

Semi-supervised learning algorithms leverage the latent structure within unlabeled data to enhance learning efficiency, reduce prediction error, and improve model generalization, often surpassing what is achievable using labeled data alone. These methods are especially valuable in non-target screening, where only a small fraction of detected features can be confidently annotated. Yet, large volumes of unlabeled data contain meaningful information about chemical similarity, transformation patterns, and instrument response behavior.

Standard semi-supervised algorithms include pseudo-labeling, semi-supervised variants of SVM, k-means, and ANN/NN architectures, co-training, and graph-based label propagation or label spreading. Although their implementations differ, these approaches typically generate or refine labels for unlabeled samples based on similarity, distance, graph connectivity, or statistical distribution patterns within the data.

A representative example is pseudo-labeling, one of the simplest and most widely adopted semi-supervised strategies. In this workflow, an initial model is trained exclusively on the labeled subset. The trained model is then used to assign provisional (“pseudo-”) labels to the unlabeled samples. Only predictions exceeding a predefined confidence threshold are incorporated back into the training dataset, after which the model is retrained. Through iterative refinement, the algorithm incrementally expands the effective training set, enabling it to capture hidden structure within the unlabeled data and improve predictive performance.

We searched Web of Science using non-target screening and machine learning within the year range from 2015 to 2025. Table 1 presents the primary machine learning algorithms employed in NTS and related HRMS workflows, along with their major application domains and usage frequencies in the literature. RF appears to be the most frequently used algorithm (30 occurrences), particularly excelling in source tracking, retention-time prediction, toxicity assessment, and structural elucidation tasks, and is also most often identified as the optimal modeling approach (10 studies). Boosting-based algorithms are the second most common (18 occurrences) and are widely used for toxicity prediction, retention-time modeling, and linking HRMS features with bioactivity. Nine studies identify them as the best-performing method. Collectively, the table illustrates that ensemble tree-based models dominate current NTS applications due to their robustness, interpretability, and strong predictive performance across a wide range of analytical tasks.

### 2.2. Workflow of NTS with Machine Learning Involved

**Figure 1:**
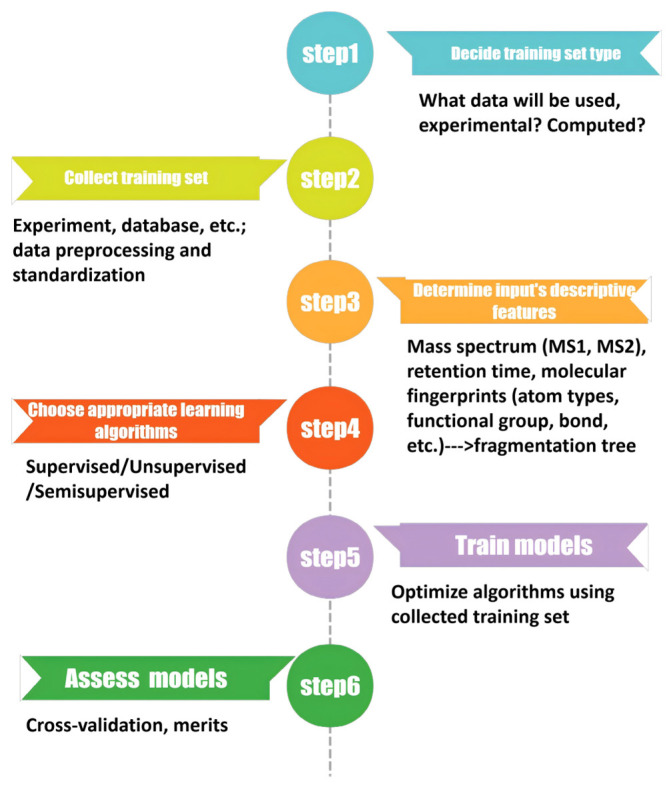
Overview of a typical machine learning workflow for non-target screening.

In supervised learning, models are trained using input–output pairs, whereas unsupervised learning extracts intrinsic structures or patterns from unlabeled data. Despite these differences, both classes of algorithms follow a series of conceptual steps when applied to NTS or mass spectrometry-based studies. A generalized ML workflow typically involves the following stages (Figure 1).

#### 2.2.1. Defining the Learning Objective and Data Type

The first step in developing an ML workflow is to clearly define the type of relationship the model is expected to learn. This requires specifying both the prediction objective and the nature of the input–output data. For instance, predicting CCS values necessitates selecting an appropriate and method-consistent reference dataset (e.g., drift-tube versus traveling-wave IMS, helium versus nitrogen buffer gas), since CCS measurements are instrument- and condition-dependent [72,73]. Similarly, predicting retention time, ionization efficiency, or fragmentation patterns relies on curated training sets with high-quality experimental annotations [74]. In contrast, tasks such as grouping unknown features based on shared chemical or spectral characteristics may depend solely on unlabeled HRMS and/or IMS data, making clustering or dimensionality-reduction methods more appropriate [75]. When only a small subset of features is annotated, for example, when a limited number of compounds have known CCS or confirmed structural identities, hybrid strategies such as semi-supervised learning can leverage the substantial unlabeled portion of the dataset to improve generalization [76]. Thus, the choice of analytical objective directly determines whether supervised, unsupervised, or semi-supervised approaches should be used, as well as the model architecture, data requirements, and evaluation criteria.

#### 2.2.2. Collecting and Preprocessing the Data

High-quality experimental data are crucial for meaningful model training. Experimentally determined outputs, such as CCS values, fragmentation spectra, or retention times, are typically the most reliable training targets. Because HRMS and IMS data may exhibit errors (e.g., ±5 ppm for mass, and 5% for CCS), background noise, or retention-time drifts, preprocessing steps such as error correction, normalization, denoising, or peak alignment are often required [25]. When spectra are converted into mathematical representations (e.g., intensity vectors [23]), standardization or scaling ensures that features contribute comparably to the learning process. Along with common data preprocessing strategies in ML, linear chemometric algorithms like MCR-ALS and PARAFAC2 remain effective options for resolving mass spectral signals. Incorrect data handling can cause overfitting and reduce predictive accuracy. However, these chemometric algorithms are often used alone and not integrated into the models.

#### 2.2.3. Selecting and Constructing Meaningful Descriptors or Features

Machine learning algorithms require numerical representations of chemical structures or instrument-derived properties as inputs. These descriptors may include molecular fingerprints, physicochemical properties, topological indices, or 3D structural descriptors generated using software tools such as RDKit, PaDEL, and alvaDesc [77]. Because thousands of potentially correlated variables can describe a single molecule, dimensionality reduction or feature selection is often necessary to minimize redundancy, improve computational efficiency, and prevent model overfitting. Approaches such as PCA or LDA can be used to identify the most informative features or construct compact representations that capture the essential variance in the dataset. For example, Hirohara et al. [78] transformed SMILES strings into low-dimensional feature vectors that encode atom types, bond connectivity, and charge distributions, demonstrating that structurally meaningful embeddings can retain rich chemical information while reducing complexity.

In those NTS workflows aimed at automated classification or risk prioritization, descriptors are derived exclusively from instrumental measurements. Parameters such as mass–charge ratio, retention time, drift time or CCS, isotopic patterns, and peak intensity can serve as effective predictors. For instance, Saer et al. [25] used only HRMS-derived variables to develop predictive models for water-quality risk assessment, illustrating that instrument-based descriptors alone can support robust ML applications when structural information is limited or unavailable. Class imbalance is another common challenge in NTS datasets, especially when positive instances (e.g., confirmed pollutants) are rare relative to the vast chemical background. To address this issue, oversampling approaches such as the Synthetic Minority Over-Sampling Technique (SMOTE) or undersampling strategies can be applied to mitigate bias and improve model generalization.

#### 2.2.4. Choosing Appropriate Learning Algorithms

Algorithm selection should be matched to the dataset’s structure and the analytical objective. In NTS, nonlinear models are often preferred because HRMS and IMS data usually contain complex and high-dimensional relationships. Linear models can perform well on simple or low-dimensional datasets but often struggle with the complex, nonlinear relationships that characterize HRMS and IMS datasets. For this reason, nonlinear algorithms are more commonly applied in NTS applications. Methods such as DT, RF, gradient boosting machines (e.g., XGBoost, LightGBM), SVMs, ANNs, CNNs, and GNNs offer greater flexibility in modeling high-dimensional and nonlinear chemical relationships [79,80]. These approaches are well-suited for tasks such as CCS prediction, spectral interpretation, compound classification, and annotation of unknown features [81,82]. For unsupervised tasks, such as revealing latent structure, detecting anomalous features, or grouping chemically related signals, algorithms including PCA, non-negative matrix factorization (NMF), hierarchical clustering, DBSCAN, and k-means are widely used. These methods can uncover inherent patterns in large datasets without the need for annotated training labels, making them particularly valuable for exploratory analysis and feature prioritization in NTS.

Because no single algorithm consistently performs best across all datasets or prediction tasks, it is standard practice to evaluate multiple models and select the one that best satisfies the analytical objective, considering factors such as predictive accuracy, interpretability, computational cost, and compatibility with available data.

#### 2.2.5. Training and Validating the Model

During model training, the algorithm learns predictive or structural patterns from the input data. A critical component of this process is hyperparameter tuning, which involves optimizing algorithm-specific settings, such as tree depth and number of estimators in random forests, learning rate in boosting models, kernel parameters in SVMs, or the number of latent components in dimensionality-reduction models. Hyperparameter optimization is commonly performed using a validation subset of the training data or through cross-validation strategies such as k-fold cross-validation. These procedures aim to maximize predictive accuracy while minimizing overfitting, thereby improving the model’s ability to generalize to unseen data [83].

For unsupervised learning methods, model tuning focuses on enhancing the quality and interpretability of the learned representation, rather than improving prediction accuracy. Examples include selecting the optimal number of components in PCA or NMF, determining the number of clusters in k-means or hierarchical clustering, or adjusting neighborhood parameters in density-based methods such as DBSCAN. Proper tuning ensures that latent structures, feature groupings, or dimensionality-reduced embeddings faithfully reflect meaningful chemical or spectral relationships.

Robust training and validation are therefore essential for developing reliable ML models for non-target screening, ensuring that the learned patterns accurately reflect the underlying chemical behavior rather than noise or instrument-specific artifacts.

#### 2.2.6. Evaluating Model Performance

After training, the model is evaluated using a test set that was withheld from all stages of learning and hyperparameter tuning. This independent assessment provides an unbiased estimate of model performance and generalizability. The choice of evaluation metrics depends on the task. For classification problems, commonly used metrics include accuracy, precision, recall, F1 score, and false-positive and false-negative rates, each of which offers insight into different aspects of predictive reliability. For regression tasks, performance is typically quantified using the root-mean-square error (RMSE), the mean absolute error (MAE), or the coefficient of determination (R^2^).

For unsupervised learning, where ground-truth labels are unavailable, model quality is assessed using cluster validity indices such as the silhouette score, Davies–Bouldin index, or density-based measures. These metrics help determine whether the learned representations or clusters reflect meaningful structure within the data.

In NTS applications, evaluation often requires domain-specific validation, including the comparison of predicted versus experimentally measured values, retention times, or fragmentation spectra to confirm chemical plausibility. Such comparisons are critical because minor deviations in mass or CCS or retention behavior may indicate structural inconsistencies, incorrect feature annotation, or limitations in the model’s training data.

## 3. Machine Learning Functions Currently Used in NTS

**Figure 2:**
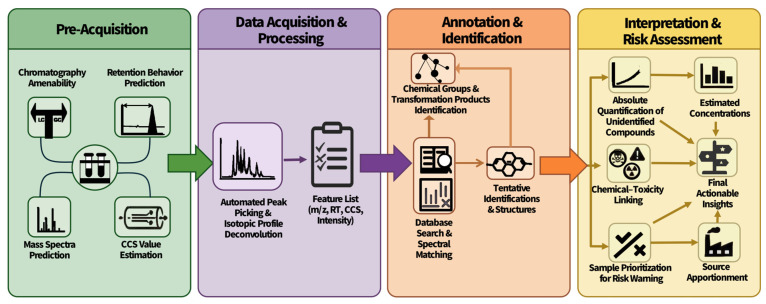
Overview of machine learning used in non-target screening process.

ML has been used in various stages of NTS, from choosing a chromatography method to instrument-derived data prediction and data interpretation, and ultimately, in toxicity for risk assessment (Figure 2). It enhances the possibility and reliability of pollutant research. Table 2 summarizes representative studies, the algorithms evaluated, their main use scenarios, and their practical limitations. The table is organized by analytical task and lists the model(s) that the references evaluated, their key practical strengths, suitable application scenarios, and major limitations. One can refer to this table for method selection and as a framework for identifying where emerging approaches may complement existing workflows.

### 3.1. Chromatography Amenability Prediction

Selecting an appropriate chromatographic separation technique, typically LC or GC, is a critical step in chemical analysis and dramatically influences the detectability and interpretability of compounds in NTS workflows. LC and GC provide complementary information beyond the *m*/*z* values obtained by MS, with GC generally favoring volatile and thermally stable compounds. At the same time, LC accommodates a broader range of polar and non-volatile substances. Traditionally, baseline predictions regarding LC or GC suitability rely on broad physicochemical characteristics such as boiling point, molecular weight, and polarity [99]. However, these rules of thumb often fail for structurally diverse or emerging contaminants.

Machine learning provides an enhanced, data-driven approach for predicting chromatographic behavior. In the study by Alygizakis et al. [26], DT algorithms were evaluated for selecting the LC/GC method using 1446 physicochemical descriptors from the NORMAN Suspect List Exchange. Eight high-impact descriptors were identified and used to train the LCvsGC model, which demonstrated superior predictive accuracy compared with conventional baseline rules. This approach enables rapid assessment of whether new or emerging chemicals are more amenable to LC-MS or GC-MS analysis and can be incorporated into suspect and non-target screening workflows to streamline analytical prioritization [100].

More recent studies have expanded this concept to instrument amenability prediction for LC-MS analyses [27] by predicting LC-MS amenability in both positive and negative electrospray ionization modes using molecular descriptors derived from SMILES strings with RF, the resulting models provide a valuable tool for anticipating which compounds are likely to be detectable under specific ionization conditions, thereby improving method selection and enhancing the efficiency of large-scale screening efforts.

### 3.2. Prediction of Chromatographic Retention Behavior

Retention time (RT) provides valuable structural information and is widely used to support the identification of unknown chemicals in NTS workflows. However, predicting RT for new or emerging pollutants is often challenging due to the lack of authentic reference standards [60]. ML-based retention time prediction models can therefore accelerate and enhance the confidence in contaminant identification by narrowing candidate lists and facilitating orthogonal validation of proposed structures.

Traditional GC retention time prediction relies on thermodynamic retention models and fluid mechanics equations that incorporate instrument-specific parameters, such as column type and length, phase ratio, and temperature program [101], as well as Quantitative Structure–Property Relationship (QSPR) models [85] and the retention behavior is often described as retention index which is a normalized retention parameter calculated relative to reference compounds. When ML algorithms are used to augment or replace these classical approaches, prediction accuracy often increases substantially [60]. For example, a multiple linear regression (MLR) model developed for several thousand compounds present in tobacco products used 20 out of 2489 molecular descriptors and achieved excellent agreement with experimental values. When integrated with QSAR-based software, the combined approach provided higher confidence in the GC-MS non-target screening of volatile and semi-volatile constituents in tobacco aerosols [85]. More recently, Vrzal et al. developed DeepReI, a CNN model that predicts GC retention indices using only SMILES strings as input, achieving a median prediction error of 0.81% [102].

For LC, however, no universally adopted retention-index system comparable to that used in GC is currently available because LC-MS accommodates a broader diversity of chemical structures, ionization behaviors, and chromatographic modes. Unlike traditional regression models that focus on structurally related compounds, ML algorithms can successfully capture RT trends across diverse chemical groups. Among these, SVMs have been widely adopted. A comparative study [28] of RF, SVMs, and GB demonstrated that GB achieved the best performance. A proposed model for RTs of contaminants of emerging concern (CECs) demonstrated robust predictive accuracy across multiple evaluation metrics, and feature-importance analysis identified the octanol–water partition coefficient and the number of basic functional groups as the most influential descriptors [103].

Significant, curated datasets have further advanced LC-RT prediction. Using the METLIN Small Molecule Retention Time (SMRT) dataset, an annotation model [29] was developed that ranked the correct molecular identity among the top three candidates in approximately 70% of cases, demonstrating the value of RT predictions as an orthogonal filter in structure identification. ANN models developed by Barron and co-workers have contributed to the discovery of approximately 100 pollutants in the suspect screening of water samples [66,67] and effectively reduce the number of candidate structures requiring manual review [64]. A comparison [30] of DT and NN models for pesticide RT prediction showed comparable performance, and the averaged model outputs supported the tentative identification of pesticide transformation products using LC-HRMS.

Still, no universally adopted retention-index system comparable to that used in GC is currently available for LC. Thus, RT in LC remains useful mainly as a method-specific aid for annotation rather than as a universally portable identifier.

### 3.3. Mass Spectral Prediction and Structure Annotation

In this review, mass spectral prediction refers to the in silico generation of MS or MS/MS spectra from chemical structure information. Accurate spectral prediction enables the generation of theoretical reference spectra for compounds that lack experimental measurements, thereby enhancing confidence in structural annotation.

For electron ionization (EI) mass spectra, several high-performance predictive models have been developed. Studies by Wei et al. [65] and Ji et al. [45] demonstrated excellent prediction accuracy using ML-enhanced approaches that capture complex fragmentation behavior. For collision-induced dissociation (CID) spectra, one of the most widely used tools is Competitive Fragmentation Modeling-ID (CFM-ID) [86]. Although originally rule-based, CFM-ID integrates probabilistic machine learning components to model fragmentation pathways and predict MS/MS spectra, and is now commonly used in both metabolomics and environmental NTS. Another prominent approach is CSI: FingerID [87], a hybrid model that combines fragmentation-tree construction with machine learning classifiers to predict the most likely chemical structure of an unknown. CSI: FingerID has demonstrated strong performance in large-scale spectral identification challenges and is frequently integrated into automated annotation workflows.

In response to community-driven benchmarking initiatives such as the Critical Assessment of Small Molecule Identification (CASMI) competition, new models are rapidly emerging. Examples include MassGenie [88] and ChemDistiller [89], which integrate deep learning architectures for both spectral prediction and automated annotation. These models utilize extensive spectral libraries and structural databases, providing enhanced accuracy and scalability for high-throughput NTS applications.

### 3.4. CCS Value Estimation

The power of regressors in predicting continuous variables is instrumental in CCS prediction. A pipeline-based gradient boosting CCSondemand has been developed to predict travel-wave CCS values (in nitrogen) using a training set of experimental data consisting of ~200 molecular descriptors (features) and over 7000 CCS values of chemicals and their adducts from diverse groups [46]. This model achieves 93.5% accuracy, with predictions within a 3% error margin, which is of the same magnitude as the measurement error in most cases. Therefore, the predicted CCS value with high accuracy can be further used in reducing false identifications. The Zhu group has developed a series of CCS (ALLCCS [90], MetCCS [92], and LipidCCS [91]) prediction tools utilizing KNN and SVM algorithms [72]. These models use SMILES as input and include experimental CCS values of massive substances of diverse groups in their training set. DeepCCS [71], built on a CNN and using SMILES as the original input, has been tested across four different instrumental platforms and outperforms other existing models. While the demand for computing power is high to train these models, it only takes a few seconds to predict a chemical’s CCS value once the model is tuned. With the CCS prediction tools, one can add CCS value as an extra dimension in NTS, which significantly enhances the potential for discovering unknown substances. For example, the annotation tool developed by the Zhu Lab [72] can provide an initial list of identified compounds when inputting CCS and *m*/*z* values with a customized threshold. Guided by CCS values from ML models, 29 pollutants were tentatively identified in indoor dust [47]. When combined with mass defect analysis, previously unknown fluorinated compounds have been identified using an ML model trained on known fluorinated substances [93].

A recent study compiled approximately 9400 experimental CCS values from 4170 environmental organic micropollutants (OMPs) to support the development of CCS prediction tools specifically relevant to environmental OMPs [94]. This work highlighted the major analytical benefits of IMS and CCS, including increased peak capacity, removal of interfering ions, improved separation of isomers, and reduction in both false positives and false negatives. As IMS resolving power continues to improve and experimental CCS databases expand, the practicality of IMS for the analysis of environmental pollutants is expected to further increase.

Most IMS instruments are currently coupled with an LC system; therefore, it is feasible to devise a model that enables the simultaneous prediction of RT and CCS. It was implemented by Mollerup et al. [63]. The four-layer ANN-based model provided fast and accurate RT and CCS values, which are helpful in NTS. Machine learning algorithms have been employed in IMS-MS data for illegal drug classification. For example, KNN, among other algorithms, was used to classify narcotic IMS data, with KNN as the most reliable algorithm [104].

Although CCS values provide an important quasi-orthogonal descriptor for compound annotation, CCS alone remains insufficient for confident annotation in many cases. A major limitation is the inconsistency of CCS measurements across different platforms, despite the high resolving power that can be achieved with advanced technologies such as Structures for Lossless Ion Manipulations (SLIM) and cyclic IMS. The development of large-scale IMS databases is feasible in principle (e.g., comprehensive CCS libraries recently published [105,106,107]), but will require substantial advances in standardized acquisition protocols, calibration strategies, database coverage, and interlaboratory reproducibility.

### 3.5. Automated Peak Picking and Isotopic Profile Deconvolution

**Figure 3:**
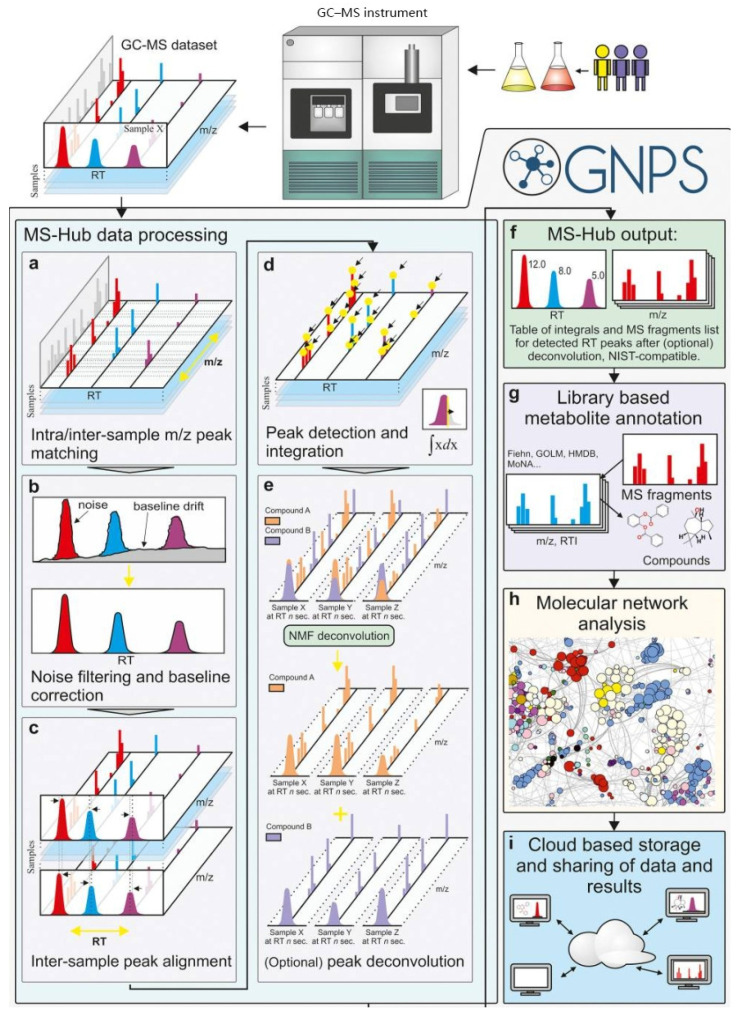
Workflow of MSHub. (**a**) Spectra were aligned, binned, denoised, and (**b**) baseline-corrected, followed by (**c**) RT alignment using Fast Fourier Transform-accelerated correlation. (**d**) Peak integrals and common fragmentation patterns were then generated, (**e**,**f**) overlapping peaks were resolved by NMF, and (**g**) the resulting features were annotated with public or private libraries, (**h**) assembled into molecular networks, and (**i**) shared with users. Reproduced with permission [97].

This is a crucial step for fully exploring the dataset generated by mass spectrometry and discovering non-targeted organic pollutants. Machine learning-guided peak picking can significantly reduce the time consumed compared with traditional manual selection, and in the meantime, capture much more information than a human being can handle. Additionally, a Windows application for ethylene-vinyl acetate analysis was developed to distinguish true peaks from false ones and to minimize false-positive identifications in LC-MS data processing. This tool employs a CNN to classify extracted ion chromatogram (EIC) peaks as true or false metabolic features [70]. A training dataset comprising 25,000 manually annotated EIC peaks from diverse sample types and LC-MS configurations was used to develop the model, enabling the resulting CNN to generalize across instruments from different vendors. Another notable feature of the software is its ability to extract peak characteristics under various processing parameters, such as mass tolerance, MS slice width, minimum peak height, and minimum peak width, demonstrating the tool’s broad applicability across different data-processing settings. There are other ML models for specific chemical groups as well [31]. IodoFinder, trained by RF using MS-MS spectra, can identify iodinated disinfection byproducts (I-DBPs) from LC-MS/MS data in both positive and negative ionization modes, overcoming the limitation of traditional methods that rely on the characteristic I^−^ fragment, which is only observable in negative mode. When applied to real DBP mixtures, IodoFinder successfully identified 19 structurally annotated and 17 formula-assigned I-DBPs, including 12 novel and 3 confirmed ones [32]. Libraries of EI spectra at 70 eV are readily available for annotating EI GC-MS spectra. However, challenges still exist, such as ununified data formats, manual parameter setting, and high operator requirements. A well-established software, MSHub (Figure 3) [97], bypasses these challenges and allows for the auto-deconvolution of GC-MS data. It was built using a one-layer NN with GC-MS descriptors. The model performs excellently across different instrument platforms and can be trained against both public and homemade libraries.

WiPP [95] is an SVM-based peak-picking pipeline that automatically detects peaks from GC-MS data. This model starts with peak separation based on intensities and annotates peaks according to their retention index and spectral similarity, within predefined thresholds. It demonstrates comparable results to manual picking for peaks of high to moderate intensities, but is less reliable for low-intensity peaks. More interestingly, it has discovered features that specialists overlooked. The deconvolution procedure has not been considered yet. Although compound identification is not the function of WiPP, it can create outputs for library search, potentially facilitating the NTS. CINeMA.py [33] (Classification Is Never Manual Again) is an ML model that automates GC×GC/TOF-MS data interpretation. It provides three algorithms, namely RF, SVM, and ANN, so that the users can choose the one with the best prediction accuracy based on their own needs.

Machine learning has also been applied to LC-MS spectra peak picking. apLCMS [34] has built in a module that enables differentiation between true peaks and noise in the EICs. The model was constructed using previously known information and a machine learning approach, with features derived from LC-MS data of known metabolites. This proof-of-concept research can be easily transferred to contamination screening.

ML has also been used indirectly in the elucidation of HRMS data. Abrahamsson et al. developed an integrated MS-ML approach for non-targeted structure elucidation, using mass spectrometers (peak areas) to measure solvent–water partition ratios as physicochemical fingerprints. A pretrained neural network converts these fingerprints into molecular fragments, offering a novel, standard-free layer of evidence for compound identification [62].

The isotopic ratio is not only crucial for element composition determination, but also capable of aiding in chemical group assignment. Chlorinated and brominated contaminants are of great concern due to their resistance to degradation. Many chlorinated and brominated contaminants have not yet been discovered or investigated [108]. Previous studies have shown that naturally occurring isotopes can form a compositional space that can guide the identification of POPs [109]. An isotopic profile deconvoluted chromatogram (IPDC) screening approach aided by an ML classifier has been reported to screen data obtained from atmospheric pressure chemical ionization (APCI) GC-MS by isolating relevant chlorinated and/or brominated compounds [48]. This algorithm was able to tentatively identify 313 and 855 halogenated features in positive and negative modes, respectively, in lake trout. In this contribution, spectrometric and chromatographic variables, such as *m*/*z*, intensity, and retention time, were utilized to develop the ML algorithm, which minimized false positives. Since APCI-GC-MS utilizes a soft ionization technique and results in little fragmentation, and thus provides molecular information, it can be a promising instrument in NTS [110]. The IPDC strategy can be used for retro analysis, taking full advantage of the extensive and comprehensive MS data [31].

### 3.6. Absolute Quantification of Unidentified Compounds

Most NTS are qualitative analyses due to the lack of authentic standards. Palm and Kruve [35] employed electrospray ionization LC-HRMS descriptors for the quantification of compounds without a previously known structure to build an ML model that predicts response factors, utilizing the RF algorithm. Response factors can be further converted to concentration, and thus, quantification is achieved. These descriptors include ratios between peak areas in positive and negative modes, *m*/*z* values and their parity, retention times and their differences under different pH conditions, and adduct types. The tuned model’s accuracy exceeds that of baseline models.

The model developed by Baek et al. [36] demonstrated that deep learning and machine learning can replace the isotopic internal standards by using the natural organic matter background in water samples. This approach could be applied to other matrices in NTS, reducing the cost of internal standard purchase or synthesis.

### 3.7. Chemical Groups and Transformation Products Identification

Chemicals of the same groups and transformation products (TPs) often share structural and physicochemical similarities with their representative or precursor compounds, and their presence in complex environmental matrices cannot be overlooked. However, traditional target screening (TS) typically detects only a limited subset of compounds. It depends heavily on prior knowledge of chemical structures, making it challenging to capture the diverse range of groups and TPs formed in the environment [49].

ML-powered (XGBoost) Pseudo-Target Screening (PTS) for Emerging Contaminants was developed to identify ECs without authentic reference standards. Tetracycline MassBank mass spectra were used as a training set, and 100% accuracy was achieved. The model demonstrates generalizability by identifying both known and novel tetracyclines [37].

Han et al. conducted suspect and non-target screening of environmental samples, followed by targeted analysis, and applied the XGBoost approach due to its strong generalizability in predicting the predicted no-effect concentration (PNEC) values of antibiotics and their transformation products, including previously unknown metabolites. The developed model was able to quantify precursor compounds in water samples and successfully detect associated TPs, demonstrating the feasibility of integrating MS/MS data with machine learning to achieve non-target prediction of unknown substances and their transformation products [49].

To identify the actual chemical drivers of acetylcholinesterase (AChE) inhibition in Chinese estuaries, Huang et al. developed a model trained across a vast chemical space, and generated a prioritized suspect list and then used this list to guide HRMS screening of water samples, which identified 60 chemicals explaining 82.1% of the observed AChE inhibition, successfully uncovering polyunsaturated fatty acids as the unexpected drivers for neurotoxicity [50].

Compared with MS alone, integrating IMS with MS can reduce interference and yield cleaner spectra. In PFAS screening, for example, the incorporation of CCS into homologous-series searching filtered out 63% of false positives, and together with RF-predicted CCS values, enabled the non-target identification of 45 additional PFASs outside the standard list [111]. This example illustrates the considerable potential of IMS-MS to enhance pollutant identification in complex environmental matrices.

### 3.8. Chemicals and Their Toxicity Correlations

Toxicity is a critical parameter in assessing the potential of organic chemicals to behave as POPs. Traditional statistical approaches are practical when toxicity is driven by a limited number of variables [112]; however, real-world environmental exposures consist of complex mixtures, making it challenging to uncover meaningful associations using classical models alone. Machine learning is increasingly applied to toxicology because it can capture nonlinear relationships and interactions among numerous environmental contaminants.

For example, ML techniques have been used to investigate the association between POP exposure and human health outcomes. Several POPs, including octachlorodibenzofuran, cis-heptachlor epoxide, PCB-77, and trans-nonachlor, were identified as strongly associated with deep endometriosis using ML-based analyses [51]. Five algorithms (regularized MLR, ANN, SVM, GB, and PLS) were compared, and all demonstrated strong capability in detecting highly correlated exposure biomarkers within complex exposure matrices. Unlike previous studies focusing on a single pollutant or chemically related groups, this analysis simultaneously evaluated multiple structurally diverse contaminants, demonstrating the feasibility of ML for toxicity assessment even when the chemical structures of contaminants are unknown or only partially characterized.

ML has also been applied to predict genotoxicity associated with organic pollutants generated during solid waste incineration [52]. These models use molecular descriptors or spectral features to infer toxicological potential, offering a rapid screening tool when experimental toxicity testing is impractical.

Bridging HRMS and bioactivity addresses the challenge of toxicity prediction in complex environments where structure determination is difficult. Additional advances in ML-based functional group classification provide complementary frameworks for toxicity prediction. LDA has been used to distinguish drug targets and identify binding sites (functional groups) in peptides and proteins using MS data [53], while CNN-based models [78] can detect both known and previously uncharacterized functional motifs. These developments suggest that unknown chemicals may be inferred by identifying proteins with characteristic binding-site patterns. For example, fluorinated organic substances are known to bind strongly to specific serum proteins [54]; Thus, ML-enabled protein-binding analysis may help flag previously unrecognized fluorinated contaminants. Electron ionization mass spectra of organic chemicals have also been linked to toxic endpoints through machine learning and experimentation, without requiring the identification of their chemical structures, with XGBoost and Random Forest models achieving high predictive accuracy [38]. Another similar example includes using MS2 fragmentation spectra to predict odor sensory attributes of chemicals [39] and the toxicity of unknown substances [40] in water and hazard-driven prioritization of features in complex aquatic environments [41], addressing the challenge of identifying odorants in complex water samples, bypassing the need for molecular structure determination.

IMS-derived descriptors such as drift time and CCS can provide structural information, helping distinguish isomers or conformers that may exhibit different toxicological behaviors. In addition, pollutants within the same group usually appear stepwise, so when combined with fixed retention time or mass differences, improvement of classification and prioritization of potentially toxic compounds within homologs is anticipated. A recent study shows that combined IMS-MS, DFT-derived molecular descriptors, and machine learning can predict the CCS values of cucurbit [6] uril-diamine host–guest complexes and classify their binding topologies [113]. The predicted CCS values clearly separated inclusion and exclusion complexes, showing that IMS-derived structural information, together with ML, can effectively capture topology-related differences in supramolecular systems. Although this study did not focus directly on toxicity, it illustrates the broader potential of ML-based IMS processing for chemical toxicity assessment.

### 3.9. Sample Types for Risk Warning Discrimination

In contrast to source tracing, hazardous factor monitoring is used to identify potential pollutants that may trigger a risk warning. Research combining ML (PCA and PLA involved) with non-targeted LC-HRMS analysis demonstrates great potential of ML in detecting xenobiotic pollutants [25]. In their study, 16 labeled chemicals were spiked into drinking water; the peak information was preprocessed via peak picking and alignment, background variability correction, and standardization. The established models can distinguish between spiked and original water samples and identify all the spiked chemicals in the test set; therefore, a risk warning system can be built once a threshold is set. Although only 16 chemicals were tested, this research sets the stage for future studies on comprehensive pollutant investigation on a more complex matrix.

Another example is DoPP, a graphical application designed for the rapid species identification of psychoactive plant materials and quantification of their psychoactive components using Direct Analysis in Real Time–High Resolution Mass Spectrometry. A fusion of RF, KNN, and SVM was trained, achieved 98% accuracy in 10-fold cross-validation and 99% in external validation [55].

In addition to MS-only approaches, IMS-enhanced workflows are increasingly being combined with ML to provide richer multidimensional information for sample classification. The successful classification of juices from different raw materials by SVM and RF models [114], together with the 100% classification of olive oil according to geographical origin by RF models [115], demonstrates the strong potential of ML-based IMS-MS data processing as an effective tool for sample characterization and discrimination. These findings further suggest that such strategies may be transferable to the differentiation of complex environmental samples.

### 3.10. Chemical Source Recognition

Classification algorithms can automatically assign samples to predefined categories, making them powerful tools for tracing the origins of chemicals in environmental or industrial contexts. HRMS datasets contain rich chemical fingerprints that reflect the composition of the injected samples, and machine learning can exploit these patterns to infer potential sources of contamination or material origin. Nonlinear tree-style algorithms, such as DT and RF, are particularly effective for source-tracing applications because they can model complex nonlinear relationships and provide interpretable information on feature importance. For example, Christopoulos et al. [23] employed a random forest classifier to categorize ~20 aerosol types based on data from single-particle mass spectrometry. Their approach successfully identified characteristic chemical markers and improved source attribution.

Similarly, Li et al. [56] applied RF models to differentiate the potential sources of virgin and recycled poly(ethylene terephthalate) (PET). In their study, more than 1200 volatile organic compounds (VOCs) detected by two-dimensional GC–MS were used as input features. The model achieved 100% classification accuracy when evaluated on additional PET batches. Analysis of variable importance revealed that high-contributing VOCs differed markedly between PET materials produced in China versus those made elsewhere, demonstrating the capacity of ML to differentiate plastics based on manufacturing or recycling origin. In the context of water-quality assessment, a related model [68] was developed to diagnose contamination sources using HRMS-derived chemical fingerprints. Remarkably, the authors found that only ten non-targeted chemical features were sufficient to discriminate among pollution sources, highlighting the potential of ML to simplify source attribution while maintaining high diagnostic accuracy.

Algorithms such as SVM and MLR have also been used in Li et al.’s work in PET source discrimination [56]. The constructed SVM models performed comparably to RF, achieving similarly high accuracy in predicting the origins of PET materials. At the same time, the result of MLR is inferior to theirs but still produces over 90% prediction accuracy. The flaws in prediction can be attributed to overfitting, which can be avoided by removing VOCs with little contribution [56] from the input data. Dávila-Santiago et al. used support vector classification to predict the presence of specific environmental sources. The method selected the top feature sets (e.g., *m*/*z*, retention time) that achieved 92–100% cross-validation accuracy in discriminating between five sources (e.g., wastewater, agricultural runoff). When applied to creek samples, the model successfully detected wastewater downstream of a treatment plant and demonstrated in simulations the ability to trace sources even at 10,000-fold dilutions [68].

Other algorithms have also been applied to source-tracing problems [23,57]. In a recent study, partial least squares discriminant analysis (PLS-DA) was used to classify intrinsic chemical signatures in groundwater samples collected from four pollution sources: oil-contaminated sites, aquaculture farms, industrial zones, and landfills [116]. By combining PLS-DA with ML-based feature selection, the authors identified the most discriminative compounds associated with each source and achieved strong classification performance, demonstrating the utility of hybrid chemometric–ML approaches for environmental forensics.

Pollutant monitoring can also require capturing temporal dynamics in addition to spatial variability. A CNN model, TrendProb [117], was trained on 30-day LC–MS NTS time-series data from river water and was able to distinguish between persistent pollutant trends and short-term events such as rainfall-driven perturbations. This study illustrates the potential of deep learning to disentangle complex temporal patterns that are not easily resolved by traditional statistical techniques.

Unsupervised ML methods can also support source classification through clustering. For example, non-target HRMS data have been used to differentiate roadway runoff from wastewater influent in urban receiving waters [9]. HCA, as applied in this study and others (e.g., Davila-Santiago et al.), helps assess the inherent separability of chemical fingerprints prior to supervised model development. However, because source categories are typically known in source-tracing applications, supervised algorithms usually outperform purely unsupervised methods once labeled training data are available.

Combining supervised and unsupervised algorithms has shown strong potential for complex classification tasks. Although the environmental distribution of micro- and nanoplastics remains analytically challenging, related work offers promising analogies. For example, ML models developed for bacterial species identification from mass spectra achieved high accuracy despite most spectral peaks remaining unassigned. By integrating PCA for dimensionality reduction with SVM and RF classifiers, these models successfully discriminated bacterial types [118]. Such findings suggest that hybrid ML approaches may also be effective for distinguishing micro- and nanoplastic types once sufficiently high-quality spectral data are acquired.

IMS-derived fingerprints can also support source apportionment in complex matrices. Using GC-IMS data, ML models successfully differentiated both the presence and type of ignitable liquid residues in fire debris, with RF achieving 100% accuracy for both residue detection and liquid-type classification [119]. Although this example is outside environmental contaminant screening, it highlights the broader potential of IMS for source-related classification in pollutant NTS workflows.

## 4. Limitations and Challenges

Machine learning is becoming more useful for mass spectrometry-based non-targeted screening, but several limitations still restrict its broader and more reliable use. Table 2 highlights the limitations, one of which is poor generalizability. Many published models are trained on relatively narrow chemical domains, specific analytical platforms, or carefully curated datasets. As a result, performance often declines when these models are applied to compounds, matrices, or instrumental conditions that differ from those represented in the training data.

A second major challenge is data quality and consistency. In workflows such as peak picking, isotope deconvolution, CCS prediction, and semi-quantification, model performance is strongly influenced by preprocessing quality, annotation accuracy, class balance, calibration strategy, and matrix effects. In addition, analytical data are not always stable across laboratories or experimental settings. Retention behavior, ionization response, spectral patterns, and CCS values may shift with instrument type, operating conditions, or data-processing choices. Such data drift can substantially reduce prediction reliability when a model is transferred beyond the dataset on which it was developed.

Interpretability also remains a practical concern. Models for source apportionment, toxicity prioritization, and bioactivity linkage can be highly useful for screening, but their outputs are often indirect and still require experimental confirmation. This issue becomes even more important in LC/GC-(IM)-MS workflows, where retention behavior, spectra, and CCS provide complementary but not equally transferable evidence. Although combining these dimensions is likely to improve annotation confidence, fully integrated ML frameworks that make consistent use of all of them are still rare.

Improving practical performance will require more than better algorithms. External validation across laboratories, broader chemical coverage in training data, and routine model updating will all be important for making these methods more transferable. Greater attention should also be paid to standardized acquisition protocols, harmonized preprocessing workflows, uncertainty estimation, applicability-domain assessment, and integrated ML frameworks. Additionally, because many machine learning algorithms operate as “black boxes”, integrating explainability tools such as SHAP (SHapley Additive exPlanations) will be important for improving interpretability in predictive outcomes.

## 5. Conclusions

Recent studies show that ML can support many parts of NTS, including source discrimination, toxicity prioritization, prediction of RT, spectra, and CCS, as well as automated peak handling and annotation. Its main contribution so far has been to improve throughput and reduce the manual burden of interpreting large HRMS.

At the same time, the practical role of ML in NTS should not be overstated. Most current methods still operate downstream of conventional analytical and data-processing workflows rather than replacing them. In addition, the confident identification of truly novel compounds remains difficult, particularly when training data are limited or when models are applied outside their original chemical and instrumental domain.

For this reason, future advances in the field will depend on more than improvements in predictive accuracy alone. Robust progress will require higher-quality and more reproducible analytical data, better multimodal integration of chromatographic, spectral, and ion-mobility information, and model designs that remain interpretable when used in real screening workflows. Under these conditions, ML is likely to become most valuable not simply as a prediction tool, but as a practical framework for combining multiple lines of analytical evidence to support more reliable annotation and prioritization of unknown contaminants in complex environmental samples.

## Figures and Tables

**Table 1:** Statistics of machine learning algorithms applied in non-target screening and related HRMS workflows.

Algorithm	Usage	Frequency of Occurrence	Optimal Algorithm Frequency	Refs.
RF	Source tracking; Retention-time prediction; Toxicity prediction; Method selection; Retention-time prediction; Automatic peak selection; isotope profile deconvolution; Absolute quantification of unidentified compounds; End-to-end linkage of HRMS features to bioactivity; Toxicant or drug classification	30	10	[23,25,26,27,28,29,30,31,32,33,34,35,36,37,38,39,40,41,42,43,44]
Boost	Toxicity prediction; Retention-time prediction; Mass spectra; CCS value prediction; Automatic peak selection; and isotope profile deconvolution; End-to-end linkage of HRMS features to bioactivity; Non-target identification of parent compounds and prediction of transformation product	18	9	[28,30,34,37,38,40,41,45,46,47,48,49]
PLS	Source tracking; Toxicity prediction; Risk early warning; Mass spectra; End-to-end linkage of HRMS features to bioactivity	10	2	[38,39,40,41,43,45,50,51,52,53,54,55,56,57,58]
SVM	Source tracking; Toxicity prediction; Retention-time prediction; Automatic peak selection; isotope profile deconvolution; Absolute quantification of unidentified compounds; End-to-end linkage of HRMS features to bioactivity; Non-target identification of parent compounds and prediction of transformation products	14	2	[25,45,50,51,57,58,59]
kNN	Toxicity prediction; Retention-time prediction; End-to-end linkage of HRMS features to bioactivity; Non-target identification of precursor compounds and prediction of transformation products	10	1	[29,37,38,39,43,60,61]
ANN	Source tracking; Toxicity prediction, Retention-time prediction; CCS value prediction; Absolute quantification of unidentified compounds; End-to-end linkage of HRMS features to bioactivity; Non-target identification of precursor compounds and prediction of transformation products	12	1	[28,36,37,47,50,51,60,62,63,64]
MLP	Toxicity prediction; Mass spectra; End-to-end linkage of HRMS features to bioactivity; Retention-time prediction	6	2	[39,40,64,65,66,67]
PCA	Source tracking; Risk early warning; End-to-end linkage of HRMS features to bioactivity; ML methods within ensemble modeling	6	0	[25,42,60,68]
LDA	Source tracking	3	2	[53,57,61]
CNN	Toxicity prediction; Retention-time prediction; CCS value prediction; Automatic peak selection and isotope profile deconvolution; End-to-end linkage of HRMS features to bioactivity	10	1	[38,60,69,70,71]

**Table 2:** Summary of machine learning algorithms applied in non-target screening and related HRMS workflows.

Analytical Task	Representative Applications	Typical Input	Typical Output	Commonly Used Algorithms	Aim	Best-Use Scenario	Main Limitations
Precursor/transformation-product prediction	Predicting likely TPs of antibiotics [49] or other contaminants [44]	Precursor structure, descriptor, fragmentation, or spectral features	Candidate TPs or TP-related properties	XGBoost, RF, ANN	Expands suspect lists and prioritizes likely TPs when standards are unavailable	Best when TP space is large and reference standards are scarce	Depends strongly on training chemistry; transferability to novel classes may be limited (generalization issue)
Source apportionment	Distinguishing runoff, wastewater, and manure [21], aerosols [23], VOC [25], food [42], cells [53], crude oil [57], groundwater sources [58], and herbs [59]	Feature fingerprint, peak intensities, chemical profiles	Source class or source contribution	PCA, PLS-DA, SVM, RF, LDA	Links chemical fingerprints to likely pollution sources	Useful for mixture classification and source discrimination in monitoring studies	Interpretation can be dataset-specific, and source categories may overlap; lack of interpretability
Toxicity prediction	Predicting liver injury [38], endocrine activity [40], AChE inhibition [50], highly toxic organochlorine pollutants [51], or in vitro toxicity [41]	Molecular descriptor, fingerprint, spectra, HRMS- derived features	Toxicity class or toxicity score	XGBoost, RF, SVM, MLP, kNN, ANN, CNN	Prioritizes hazardous unknowns for follow-up testing	Useful when toxicological testing capacity is limited	Predicted toxicity is screening-level only and still requires experimental confirmation due to data availability
Method selection	Choosing LC-HRMS vs. GC-HRMS or selecting a data-analysis route for CECs [26]; Organic small molecules [27]	Chemical properties, platform metadata, feature sets	Guided analytical method or workflow	DT, RF, rule-based classifiers	Helps match analyte classes to analytical platforms	Useful during workflow design and screening strategy selection	Decision rules may not transfer across instruments or laboratories (generalization)
Retention behavior prediction	Metabolites [29,84], pesticides [30,64,67], pharmaceuticals [64,66,60], tobacco-related compounds [85], and large datasets [28]	Molecular descriptor, fingerprint, chromatographic conditions	Predicted RT or RI	SVR, XGBoost, RF, ANN, kNN, SVM, MLP, GNN	Improves annotation confidence and filters implausible candidates	Especially useful for suspect screening and candidate ranking	Highly method-specific; cross-transferability may be limited (generalization)
Early risk warning	Drinking water directly from six treatment plants [25]	HRMS feature profiles, peak intensities, and chemical fingerprint	Warning classification of abnormal samples	PLS-DA, PCA	Ensemble model detected all spiked analytes with high accuracy and precision	Useful for high-confidence early warning in monitoring	Performance under routine real-world conditions may be lower than under controlled spiking experiments (overfitting)
Mass spectral interpretation /annotation	EI-MS [45,65] or MS/MS library matching [86], fragmentation prediction, and structure ranking (VOC) [61]; iodized chemicals [32]; and small molecules [46,62,87,88,89])	Spectra, molecular finger- prints, structure encodings	Candidate structures, fingerprint, or predicted spectra	DNN, MLP, SVM, XGBoost, SVR, GNN	Improves structural annotation and ranking of unknowns	Best for large candidate spaces and library -assisted identification	Performance drops for compounds outside the training or library space (generalization issue)
CCS prediction	Metabolites [71,90], lipids [91], pollutants [47,92,93,94]	Molecular descriptor, finger- prints, structures	Predicted CCS	SVR, CNN, ANN, XGBoost	Adds an orthogonal filter for candidate screening	Especially useful in IMS- enabled workflows	Large difference across platforms (data quality issue); accuracy depends on compound class, adduct, and domain coverage (generalization)
Automatic peak selection/isotope deconvolution	Peak-noise classification [69], isotope pattern recognition, false-positive reduction (metabolites) [34,70,95,96], pollutants [32,33,43,48,31,97,98]	Raw or processed chromato- graphic and spectral signals	Peak class, isotope grouping, cleaned feature list	CNN, RF, SVM, RUSBoost, NMF, Adaboost	Improves data quality and reduces manual inspection burden	Useful for large HRMS and GC-HRMS datasets	Performance depends on preprocessing, labeling quality, and class balance (data quality, overfitting)
Quantification/classification without standards	Estimating concentration or classifying toxicants or drugs without authentic standards [35,36,37]	Spectral features, descriptor, response patterns	Estimated concentration or chemical class	RF, regularized RF, ResNet, XGBoost, ANN	Supports semi- quantification and rapid prioritization	Useful where standards are missing or too many compounds are present	Uncertainty can remain high, and matrix effects may limit accuracy (data quality, interpretability)
End-to-end linkage to bioactivity	Linking unknown features or mixtures to toxicological activity [38,39,84]	HRMS features, spectra, bioassay -linked data	Bioactivity score or activity class	XGBoost, RF, SVM, CNN, DNN, BRR	Bridges chemical data and effect-based screening	Useful for prioritizing unknowns in effect- directed analysis	Biological interpretation remains indirect and requires follow-up validation (overfitting, interpretability)

## Data Availability

No new data were created or analyzed in this study. Data sharing is not applicable to this article.
